# Safe and reliable removal of a migrated biliary metal stent with a snare placed over the balloon using the two-devices-in-one-channel method

**DOI:** 10.1055/a-2545-2440

**Published:** 2025-03-12

**Authors:** Kazuya Sumi, Jun Ushio, Hisaki Kato, Akinori Komagata, Yuki Kawasaki, Takayoshi Ito, Haruhiro Inoue

**Affiliations:** 1 Digestive Disease Center, Showa University Koto-Toyosu Hospital, Tokyo, Japan

Metal stents are useful for the palliative treatment of malignant biliary stenosis (MBS); however, they can occasionally migrate, complicating removal. Stent removal is often performed using the rat tooth forceps, snares, basket, and balloon catheter 1 2 3 , but the efficacy and reliability of these methods are limited. This video demonstrates the successful removal of a migrated stent using the two-devices-in-one-channel method, with a snare positioned over a balloon catheter.

A 55-year-old woman with obstructive jaundice caused by pancreatic head cancer underwent the placement of a metal stent and was being treated with chemotherapy. A fully covered metal stent (Φ8 mm × 6 cm; Taewoong Medical Co., Ltd, Goyang-si, South Korea) was inserted ( Fig. 1 ).

**Fig. 1:**
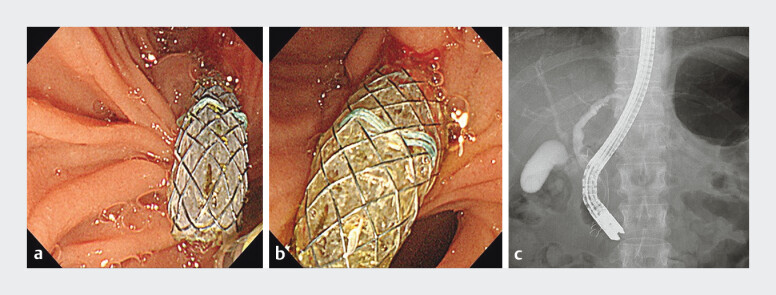
Insertion of a covered metal stent (Φ8 mm × 6 cm) into the distal malignant biliary stenosis. **a, b** Endoscopic images. **c** Fluoroscopic image.

On the day of her visit, she had epigastric pain and fever. Diagnostic evaluation revealed stent migration and cholangitis, and an emergency endoscopic retrograde cholangiopancreatography (ERCP) was performed on the same day. The migrated metal stent was positioned above the distal MBS ( Fig. 2 ). Our attempt at removing the metal stent using a balloon catheter was unsuccessful, as tumor compression impaired device maneuverability, making it difficult to control the rat tooth forceps. The procedure was temporarily discontinued after insertion of an endoscopic nasobiliary drainage tube.

**Fig. 2:**
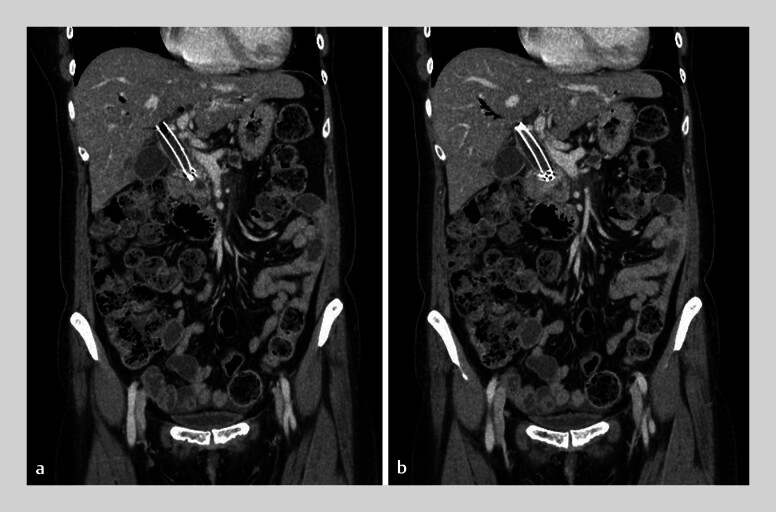
The migrated metal stent was positioned within the confluence of the hepatic duct and distal malignant biliary stricture.

During the second ERCP attempt, the snare was positioned over the balloon, and the balloon portion was inserted into the stent. Inflating the balloon within the stent enabled the reliable deployment of the snare outside the stent, where it securely grasped the stent and the balloon. Both the stent and balloon were subsequently withdrawn after deflating the balloon ( Fig. 3 , Fig. 4 , Video 1 ).

**Fig. 3:**
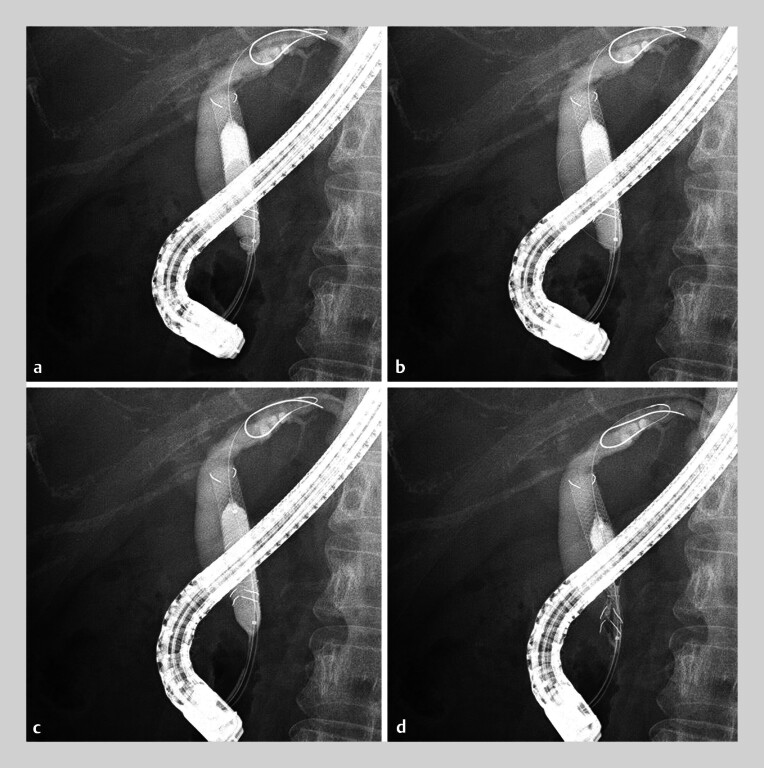
The withdrawal of both stent and balloon together by grasping with the snare.

**Fig. 4:**
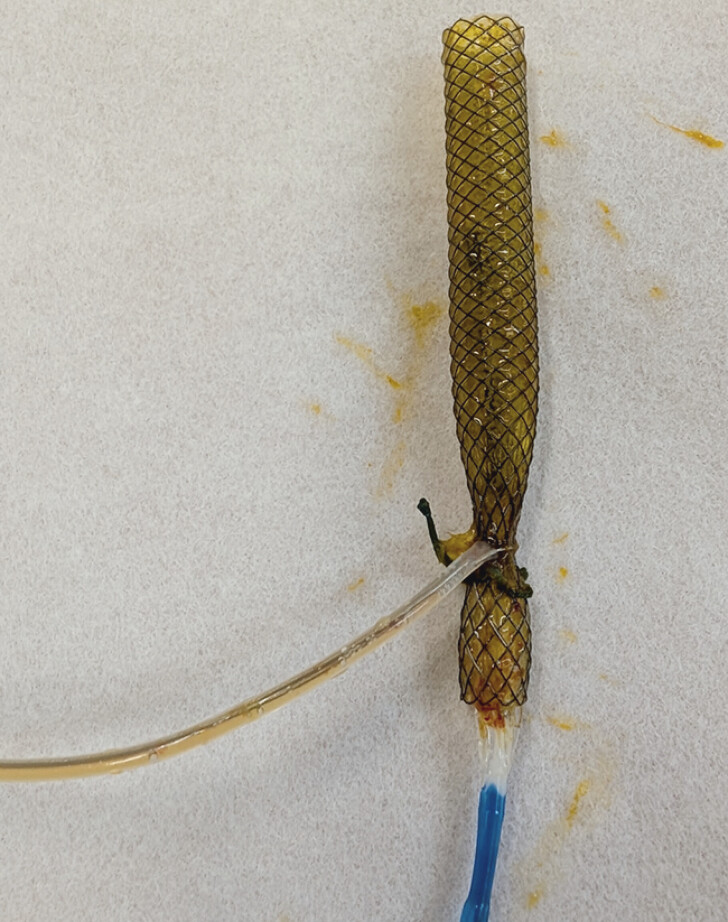
The retrieved metal stent.

A two-devices-in-one-channel method was employed for the safe and reliable removal of a migrated biliary metal stent by placing a snare over a balloon dilation catheter.Video 1

This technique employed the combined application of a balloon dilation catheter and a snare, providing a reliable method that did not require specialized devices. This approach may serve as a valuable method for stent removal.

Endoscopy_UCTN_Code_TTT_1AR_2AZ

## Notes

Endoscopy E-Videos https://eref.thieme.de/e-videos
*E-Videos* is an open access online section of the journal *Endoscopy* , reporting on interesting cases and new techniques in gastroenterological endoscopy. All papers include a high-quality video and are published with a Creative Commons CC-BY license. Endoscopy E-Videos qualify for HINARI discounts and waivers and eligibility is automatically checked during the submission process. We grant 100% waivers to articles whose corresponding authors are based in Group A countries and 50% waivers to those who are based in Group B countries as classified by Research4Life (see: https://www.research4life.org/access/eligibility/ ). This section has its own submission website at https://mc.manuscriptcentral.com/e-videos .

## References

[1] Wu C Yang J Xu M Endoscopic removal of a proximally migrated biliary metallic stent Endoscopy 2017 49 E99 100 10.1055/s-0043-101521 28192807

[2] Li MKK Sze KK Tong TYH Successful retrieval of migrated and embedded fully covered self-expanding metal stent using “snare and lithotripter” technique Endoscopy 2023 55 E390 E391 10.1055/a-2008-0320 36736358 PMC9897942

[3] Cho NJ Lee TH Park SH Endoscopic removal of a proximally migrated metal stent during balloon sweeping after stent trimming Clin Endosc 2013 46 418 422 23964344 10.5946/ce.2013.46.4.418 PMC3746152

